# Generation of T cell containing adipose tissue organoids

**DOI:** 10.1016/j.isci.2026.117429

**Published:** 2026-09-03

**Authors:** Christian Kühne, Yasmin Majlesain, Sudheendra Hebbar Subramanyam, Christopher T. Neullens, Charlotte Lahrtz, Tim Ruhl, Justus Beier, Sabrina B. Bennstein, Lothar Rink, Klaus Tenbrock, Heike Weighardt, Kim Ohl

**Affiliations:** 1 Institute of Immunology, Medical Faculty, RWTH Aachen University, Pauwelstrasse 30, 52074 Aachen, Germany; 2 Immunology and Environment, Life & Medical Sciences (LIMES) Institute, University of Bonn, Bonn, Germany; 3 Department of Pediatrics, Pediatric Rheumatology, RWTH Aachen University, Aachen, Germany; 4 Department of Plastic Surgery, Hand Surgery-Burn Center, University Hospital RWTH Aachen, Aachen, Germany; 5 Institute for Tumor Biology and Experimental Therapy, Georg-Speyer-Haus, Frankfurt am Main, Germany; 6 Division of Pediatric Rheumatology, Department of Pediatrics, Inselspital, University of Bern, Bern, Switzerland; 7 Faculty of Food and Nutrition Sciences, University of Applied Sciences, Hochschule Niederrhein, Moenchengladbach, Germany

**Keywords:** adipose tissue T cells, metaflammation, obesity, tissue organoide

## Abstract

T cells and especially regulatory T cells play a pivotal role in adipose tissue homeostasis. Peripheral blood T cells are well studied; however, in-depth analysis of adipose tissue T cells in their environment is challenging, and the analysis of adipose tissue-resident T cells is currently restricted to *in vivo* mouse models and *ex vivo* human donor samples. We developed a model to study T cells in adipose tissue by means of an adipose tissue organoid model. We could observe a clear T cell infiltration in these organoids. Interestingly, we observed different infiltration ratios of regulatory T cells and CD4^+^ effector T cells. In addition to this, mimicking the inflammatory milieu in adipose tissue by the addition of lipopolysaccharide (LPS) enhanced CD4^+^ T cell infiltration within adipose tissue organoids. In summary, we herein provide a suitable *ex vivo* tool to analyze adipose tissue-resident T cells from different fat depots under near-physiological conditions.

## Highlights

• T cells and regulatory T cells migrate in adipose tissue organoids
• The migration depends on the source of adipose tissue and can be enhanced by LPS
• Regulatory T cells reveal a distinct migration behavior from effector T cells

## Graphical abstract

**Figure:**
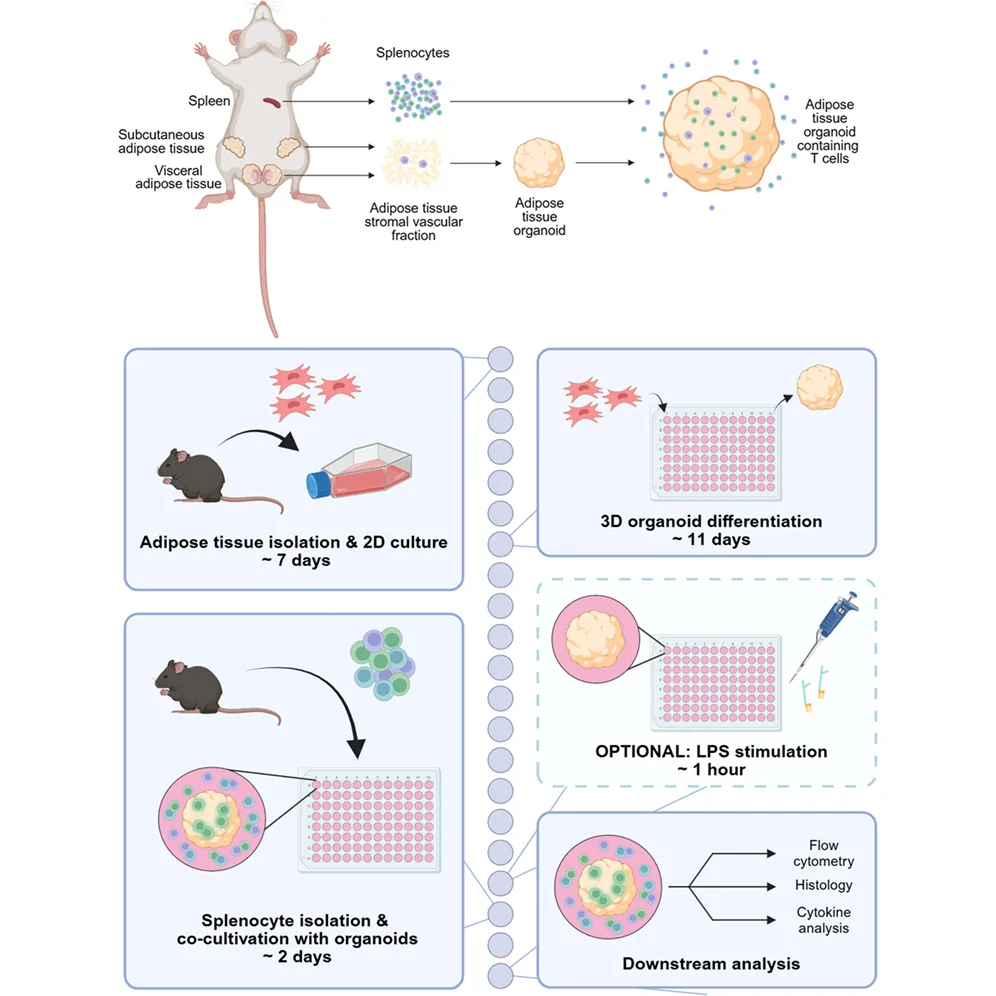

## Teaser

Immunology; Cell biology

## Introduction

Metabolic diseases, such as type 2 diabetes mellitus (T2DM), metabolic dysfunction-associated steatotic liver disease (MASLD), and cardiovascular diseases, emerge as the global health challenge of the 21st century with implications for individuals, healthcare systems, and economies.^1^ Obesity is one of the major contributors to metabolic diseases and is associated with low-grade inflammation within fat depots.^2^ Numerous immune cells including neutrophils, macrophages, B and T cells have been identified in adipose tissue. The close interaction between immune cells and adipose tissue highlights the need for in-depth research to address fundamental questions about how to target this niche-specific immune-adipose tissue crosstalk selectively and effectively.^3^

Emerging evidence suggests that regulatory T cells (T_regs_) are a decisive cell population in adipose tissue homeostasis which influences the pathogenesis of obesity-related metabolic disorders.^4^ Visceral adipose tissue (VAT)-resident T_regs_ in humans are negatively affected by obesity and have an important role in maintaining glucose tolerance.^5^ The loss of T_regs_ in *in vivo* depletion experiments exacerbates adipose tissue inflammation and alters metabolic parameters such as increased blood glucose levels accompanied by decreased insulin sensitivity.^6^ This expands the role of T_regs_, which are primarily known as regulators of immune homeostasis, toward a role for the maintenance of organismal homeostasis by interacting with adipocytes and local immunocytes. It is known that VAT T_regs_ bear a distinct transcriptional profile and antigen receptor repertoire compared with their lymphoid tissue counterpart; however, the exact mechanism by which adipose tissue T_regs_ are regulated remains less clear.^5^ Due to the low numbers of T cells that can be isolated from fat tissue, it is challenging to perform functional analysis of VAT-resident T cells and analyze T cell tissue interactions.

We therefore expanded an *ex vivo* adipose-tissue model^7^ that has been proven to be suitable for immunometabolic studies. Besides mature adipocytes, the organoids were confirmed to also contain immune cell populations, which mostly consist of macrophages and eosinophils,^7^ but not T cells. Here, subcutaneous adipose tissue (SAT) and VAT from lean mice are used to isolate adipose tissue stromal vascular fraction (SVF) cells which then undergo self-organization in spheroids. Adipocyte differentiation is then induced using a standard adipocyte differentiation cocktail consisting of insulin, dexamethasone, rosiglitazone, and 3-isobutyl-1-methylxanthin (IBMX). To study the role of T cells in adipose tissue immunology, we externally added T cells from a murine spleen to this model which results in the migration of T cells into the adipose tissue organoid.

## Results

### T cells infiltrate VAT-derived adipose tissue organoids

**Figure 1:**
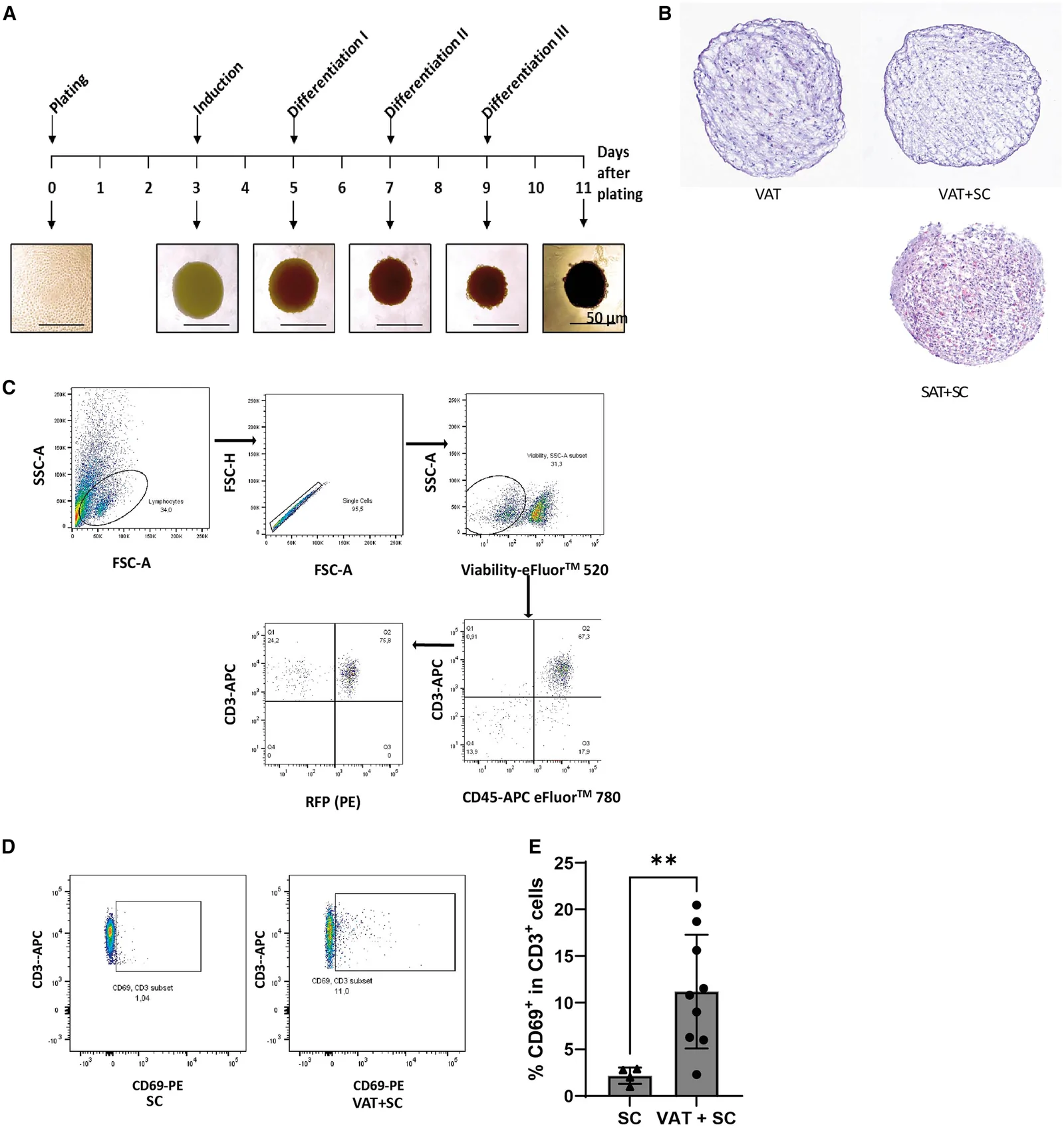
Generation of adipose tissue organoids containing T cells (A) Timeline for adipose tissue organoid generation. Adipose tissue organoids were generated by plating isolated adherent cells from the adipose SVF into coated 96 U bottom well plates. 50,000 cells were plated per well in 200 μL growth medium. The cells are cultivated at 37°C with 5% atmospheric CO_2_. After 2 to 3 days, the growth medium was replaced with induction medium. After the induction, the medium was replaced with differentiation medium on the fifth, seventh, and ninth days after plating. The organoids were used for co-cultivation with splenocytes in further experiments and analysis. Depicted are murine WT organoids generated from VAT-derived SVF. (B) Representative photomicrographs of hematoxylin and eosin (H&E)-stained organoids. (C) Gating strategy for the identification of T lymphocytes and T_regs_ in an adipose tissue organoid model. After the differentiation into adipose tissue organoids and the co-cultivation with murine SC was completed, the organoids were isolated, washed, and digested for subsequent flow cytometric analysis. Staining was performed with Viability Dye eFluor 520 (FITC), anti-mouse CD45 APC eFluor 780 (APC-Cy7), and anti-mouse CD3 APC antibodies. RFP is used as an indicator for FOXP3 expression and measured in the PE channel. Depicted is a co-culture of VAT organoids with splenocytes from FOXP3^CRE^ROSA^RFP^ mice. (D) Flow cytometric analysis of CD69 expression on C57BL/6 WT mice T cells cultured in splenocyte monoculture (SC, *N* = 4) and T cells, which infiltrated in organoids (VAT + SC, *N =* 9). Only CD3^+^ and viable cells are shown. (E) Statistical analysis of (D), a Kruskal-Wallis test was used to test for significance, followed by Dunn’s multiple-comparison test. Bars indicate mean and error bars SEM. SVF, stromal vascular fraction; VAT, visceral adipose tissue; SC, splenocytes; WT, wild type. Values were considered significant if ∗*p* < 0.05, ∗∗*p* < 0.01, ∗∗∗*p* < 0.001, and ∗∗∗∗*p* < 0.0001. *N* represents the number of biological replicates.

We established a protocol to generate murine adipose tissue organoids containing T cells within 20 days. The process includes adipose tissue isolation and 2D pre-culture, 3D organoid differentiation into adipose tissue organoids, and co-cultivation with splenocytes to introduce the T cells. Therefore, VAT was harvested from the gonadal fat surrounding the testis. SAT was isolated from the posterior inguinal tissue around the hind limbs. The 3D culture of adipose tissue organoids consists of initial cell seeding to an ultra-low attachment 96-well plate which facilitates the spheroid formation within 3 days. Adipocyte differentiation is induced by cultivating the spheroids in induction and differentiation medium for a total of 8 days. After 11 days, adipose tissue organoids are considered completely differentiated and can be used for the co-cultivation with splenocytes (Figure 1A). When analyzing spheroid structure and morphology by H&E staining, we detected small dense cells (Figure 1B). As described by us before, adipose tissue organoids contain SVF-derived macrophages but not T cells.^7^ We therefore decided to externally add T cells in this setting. To this end, fully differentiated adipose tissue organoids were co-cultivated with splenocytes to introduce T cells to the organoids. While H&E-stained sections did not show any obvious differences after T cell co-culture (Figure 1B), viable T cells migrating to and infiltrating the adipose tissue were identified by flow cytometry (Figure 1C). The enzymatic digestion before flow cytometric analysis hampered CD4 and CD8 discrimination of the CD3^+^ population (Figures S1A and S1B). We observed a slight decline of CD4^+^ T cells in the supernatant of co-cultures, while CD8^+^ T cells were not reduced (Figures S1C and S1D). This suggests that mainly CD4^+^ T cells infiltrate the organoids. However, better staining protocols are required to prove this hypothesis. CD69 expression on infiltrated T cells was enhanced compared with not-infiltrating T cells, however, just about 10% of all infiltrated T cells expressed CD69 (Figure 1D), which indicates that only a minority of these cells are in an activated state.

### T cell infiltration in SAT adipose tissue is marginal

**Figure 2:**
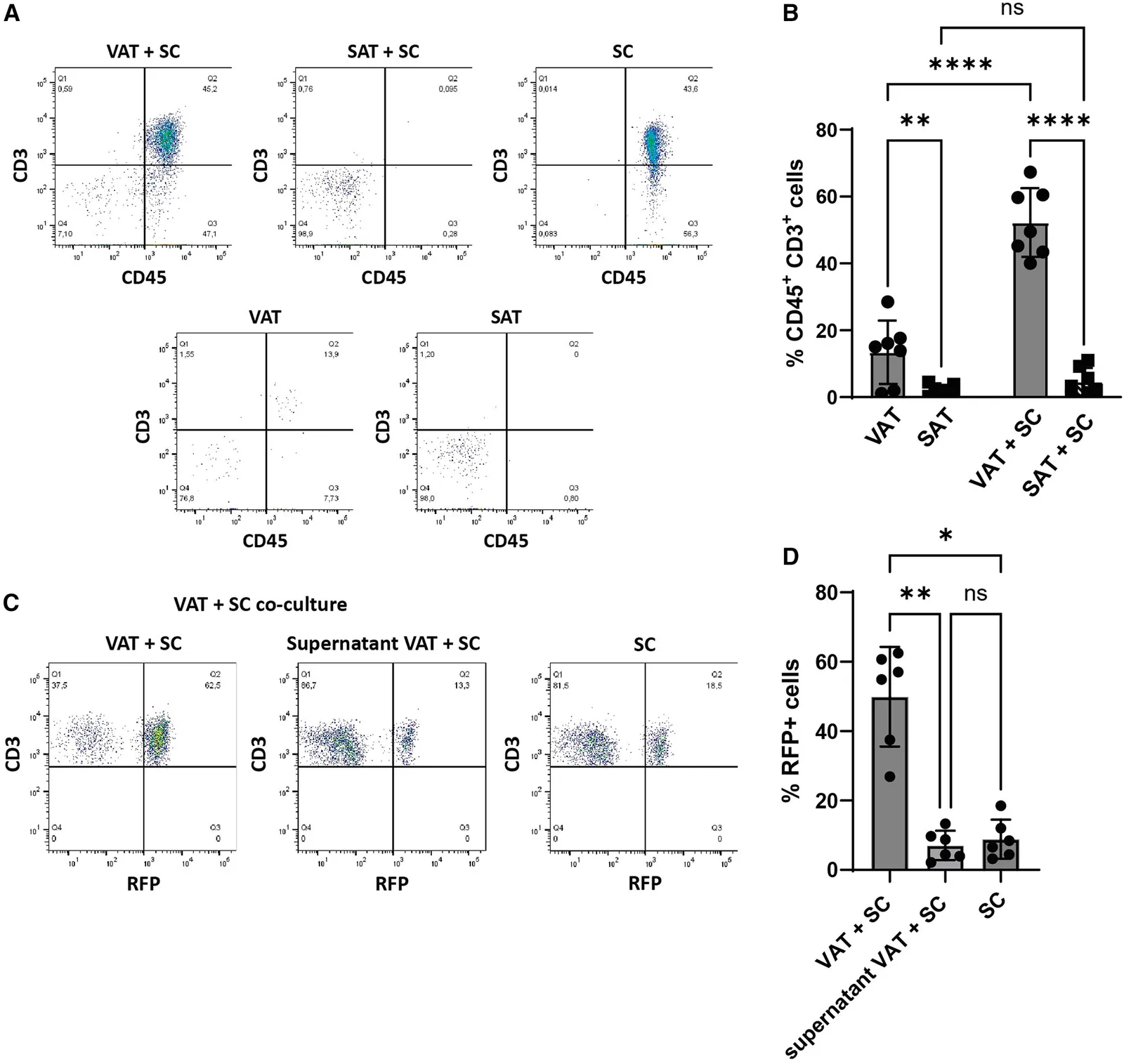
T cells infiltrate VAT organoids but not SAT organoids during co-cultivation with splenocytes (A) Representative CD45^+^ CD3^+^ T cell populations of the viable lymphocyte fraction residing in VAT and SAT organoids in monoculture or co-cultivated with SC. (B) T cell frequencies were calculated as the percentage of CD45^+^ CD3^+^ of all viable cells. FITC-negative cells are termed viable cells, therefore excluding autofluorescent and dead cells from the organoid cultures. The data were analyzed by using two-way ANOVA to distinguish between organoids from adipose tissue types (VAT vs. SAT) and co-culture setting (SAT + SC and VAT + SC). All analyses were performed on untransformed percentage values. Significant interaction effects were followed by Tukey’s post hoc tests. (C) Representative populations of T_reg_ cells in SC monoculture and in VAT + SC co-cultures from the supernatant and organoid fraction. T_reg_ cells are identified via RFP expression which indicates FOXP3 expression of cells in FOXP3^CRE^ROSA^RFP^ mice. (D) T_reg_ cell frequencies were calculated as percentage of CD45^+^ CD3^+^ T cells expressing RFP (FOXP3). For statistical comparison of the T_reg_ frequencies, a non-parametric Kruskal-Wallis test was used followed by Dunn’s multiple-comparison test. Fully differentiated VAT and SAT organoids were co-cultivated with SC from FOXP3^CRE^ROSA^RFP^ mice or kept in monoculture. SC monoculture was prepared as control. After co-cultivation, the organoids were isolated, washed and digested for flow cytometric analysis. Surface staining was performed with Viability Dye eFluor 520 (FITC), anti-mouse CD45 APC eFluor 780 (APC-Cy7) (1:100), and anti-mouse CD3 APC antibodies. RFP expression is used to identify T_reg_ cell populations. For each sample, four organoids were used. For the SC sample, 1 Mio. cells were analyzed. Seven replicates were measured per sample. GraphPad Prism (v10.6.1.) was used for all analyses. Data are presented as mean ± SEM and the indicated data points*, N =* 6. Statistical significance is represented as ∗*p* < 0.1, ∗∗*p* < 0.01, ∗∗∗*p* < 0.001, ∗∗∗∗*p* < 0.0001, or ns (not significant). Ctrl, Control; RFP, red fluorescent protein; SAT, subcutaneous adipose tissue; SC, splenocytes; VAT, visceral adipose tissue.

Subsequently, we tested T cell infiltration in our adipose tissue organoid model in different settings and performed cell analysis by flow cytometry to analyze the frequencies of adipose tissue organoid infiltrating T lymphocytes. The cell surface markers CD3 and CD45 were used to identify T cells within the different cultures (Figures 2A and 2B). Splenocytes (SCs) and organoids cultivated separately under the same conditions were used as control. Interestingly, we found that T cells are present in VAT organoids but not in SAT organoids in unstimulated cocultures (Figure 2B). To analyze, T_reg_ cells in the organoid co-culture, we made use of Forkhead box P3 (FOXP3) reporter mice to monitor T_reg_ cells without the need of intracellular FOXP3 staining. The T cell population was analyzed for red fluorescent protein (RFP) expression to identify T_reg_ cells (Figures 2C and 2D). Interestingly, T cells within the VAT organoids demonstrated an increased ratio of T_reg_ cells in comparison with the T cells in the co-culture supernatant (Figure 2D), which might indicate that preferably T_reg_ cells infiltrated VAT organoids.

### Lipopolysaccharide stimulation induces inflammation in adipose tissue organoids and induces T cell infiltration in SAT-derived adipose tissues

**Figure 3:**
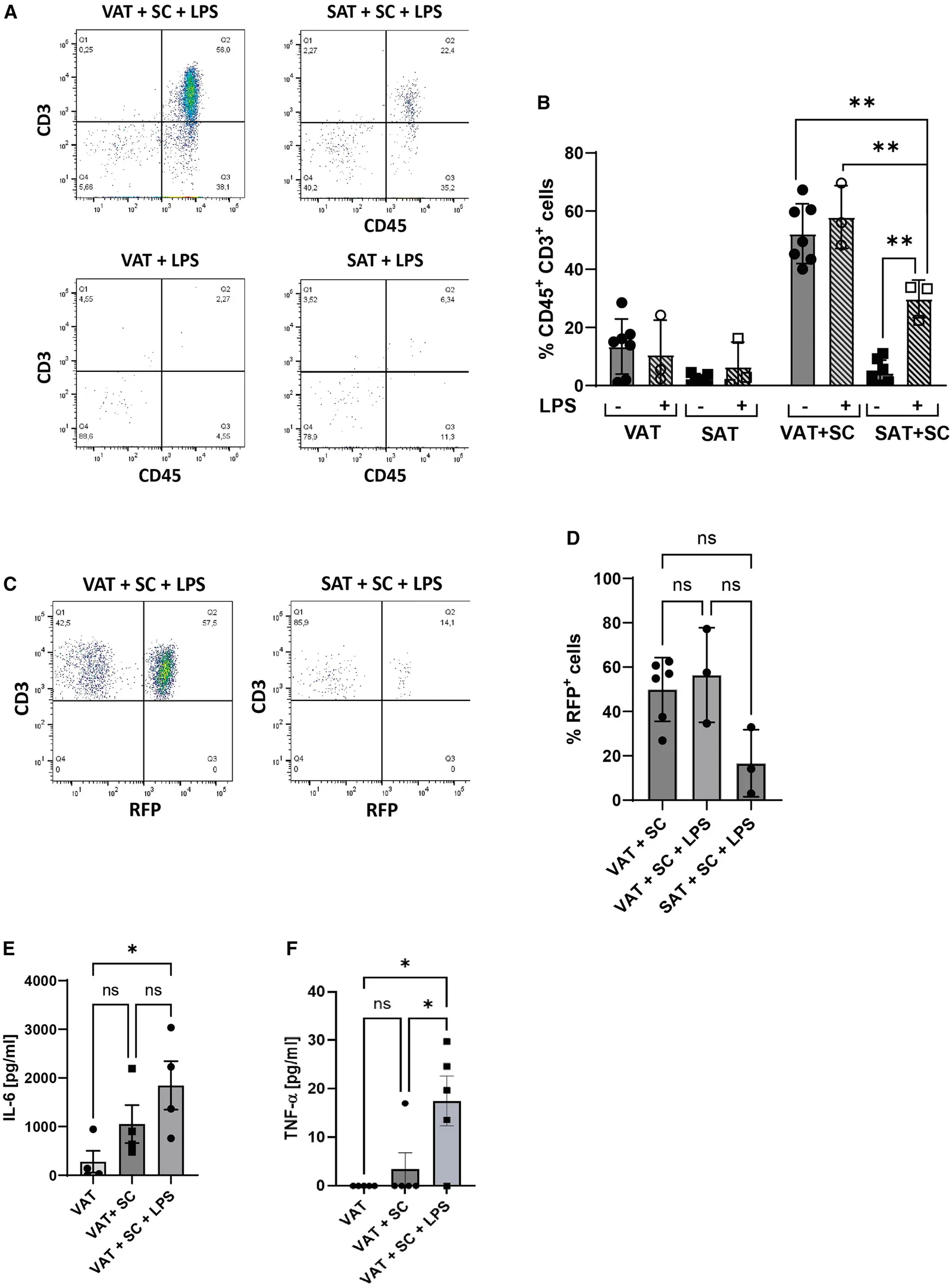
LPS stimulation induces T cell infiltration into VAT and SAT organoids (A) VAT and SAT organoids were stimulated with 10 ng/mL LPS for 1 h at 37°C. After stimulation, the samples were either co-cultivated with SC from FOXP3^CRE^ROSA^RFP^ mice or remained as monoculture. After cultivation for at least two days, the samples were isolated, digested and prepared for flow cytometric analysis. Surface staining was performed with Viability Dye eFluor 520 (FITC) (1:1,000), anti-mouse CD45 APC eFluor 780 antibody (APC-Cy7), and anti-mouse CD3 APC antibody. For each sample, four organoids were used. Three replicates were measured per sample. (B) T cell frequencies were analyzed using a three-way factorial ANOVA with organoid type (VAT vs. SAT, *N=7*), co-culture condition (VAT + SC, SAT + SC, *N =* 7), and LPS stimulation (− vs. +, *N =* 3) as fixed factors, post hoc pairwise comparisons were performed with Sidak’s correction for multiple testing. (C) Representative populations of T_reg_ cells in the organoid fraction of LPS-stimulated VAT + SC and SAT + SC co-cultures. T_reg_ cells are identified via RFP expression which indicates FoxP3 expression in FOXP3^CRE^ROSA^RFP^ mice. (D) T_reg_ cell frequencies were measured as percentage of CD45^+^ CD3^+^ T cells expressing RFP (FoxP3). For statistical comparison of the T_reg_ frequencies, a non-parametric Kruskal-Wallis test was used followed by Dunn’s multiple-comparison test (*N =* 3–6). (E) Determination of IL-6 concentrations in the supernatant of VAT organoid cultures without splenocytes, with splenocytes, and with splenocytes and LPS stimulation by ELISA *(N =* 4), a two-tailed, unpaired *t* test was performed to test for significance. (F) Determination of TNF-α concentrations in organoid cultures without splenocytes, with splenocytes, and with splenocytes and LPS stimulation by ELISA (*N =* 4), a two-tailed, unpaired *t* test was performed to test for significance. GraphPad Prism (v10.6.1.) was used for all analyses. Data are presented as mean ± SEM and the indicated data points. Statistical significance is represented as ∗*p* < 0.1, ∗∗*p* < 0.01, ∗∗∗*p* < 0.001, ∗∗∗∗*p* < 0.0001, or ns (not significant). Ctrl, Control; IL-6, interleukin 6; LPS, lipopolysaccharide; SAT, subcutaneous adipose tissue; SC, splenocytes; TNF-α, tumor necrosis factor α; VAT, visceral adipose tissue.

Next, lipopolysaccharide (LPS) stimulation of the adipose tissue organoids was performed to simulate a pro-inflammatory environment mimicking adipose tissue inflammation. The effect of LPS on T cell infiltration into adipose tissue organoids was analyzed via flow cytometry (Figure 3A). LPS treatment slightly, however, not significantly increased T cell infiltration into VAT organoids. To further confirm that the infiltrating T cells mainly consist of CD4^+^ T cells as suggested from our results in Figures S1C and S1D, we additionally performed intracellular staining for CD4 and CD8. This experiment revealed that mainly CD4^+^ T cells enter VAT after LPS exposure, while CD8^+^ T cells remained in the supernatants (Figures S2A and S2B). Interestingly, LPS stimulation resulted in an increased infiltration of T cells into co-cultivated SAT organoids (Figures 3A and 3B). However, the ratio of infiltrating T cells was lower compared to VAT organoids. LPS stimulation of co-cultivated VAT organoids may also enhance T cell infiltration (Figure 3B). Flow cytometric analysis also indicates a lower T_reg_ ratio in LPS-stimulated SAT organoids compared with its counterpart in VAT organoids (Figures 3C and 3D). Interleukin 6 (IL-6) and tumor necrosis factor α (TNF-α) concentrations were measured by ELISA in supernatants from VAT organoids in monoculture, VAT organoids co-cultured with splenocytes and LPS-stimulated co-cultures to determine the secretion of inflammatory cytokines. Interestingly, co-cultivation with splenocytes tendentially enhanced IL-6 secretion which further increased upon LPS stimulation (Figure 3E). TNF-α secretion increased significantly upon LPS stimulation in VAT organoid co-cultures (Figure 3F).

## Discussion

Our proof-of-concept study revealed interesting findings that increase the physiological relevance of adipose tissue organoids to answer further scientific questions. In our co-culture, we could only observe T cell migration into VAT organoids but not SAT organoids. Under lean conditions, *in vivo* experiments showed that VAT contains a distinct T_reg_ population, which declines with obesity.^8^ During obesity, the high release of inflammatory cytokines is primarily associated with VAT. Excessive VAT and the subsequent release of inflammatory cytokines is associated with an increased mortality and a higher risk for metabolic diseases. The infiltration of CD4^+^ effector T cells into VAT results in a shift from an anti-inflammatory milieu to a pro-inflammatory environment which drives initiation and propagation of adipose tissue inflammation.^9^ We also observed a higher IL-6 release in LPS-stimulated VAT adipose tissue organoids. This suggests that our model mimics physiological inflammatory processes in VAT *in vivo*. In line with this, we did not observe any infiltration in brown adipose tissue (BAT) (data not shown). The consistently high infiltration rates of T_reg_ cells in unstimulated VAT organoids are unexpected, and further studies should be performed to characterize their phenotype and function within this model. Additional characterization of the infiltrating T_reg_ cells is necessary to clarify whether these T_reg_ cells do adequately resemble bona fide adipose tissue T_reg_ cells. Also, we hypothesize that our unstimulated VAT adipose tissue model more resembles lean adipose tissue. Quite recently, Elkins et al. showed that cholesterol synthesis is a key metabolic pathway for VAT T_reg_ accumulation and depletion of VAT T_regs_ in obese humans and mice could partially be caused by a disruption of cholesterol homeostasis-mediated clonal expansion in VAT T_regs_. It might be interesting to analyze this in detail in our organoid model.^10^

**Figure 4:**
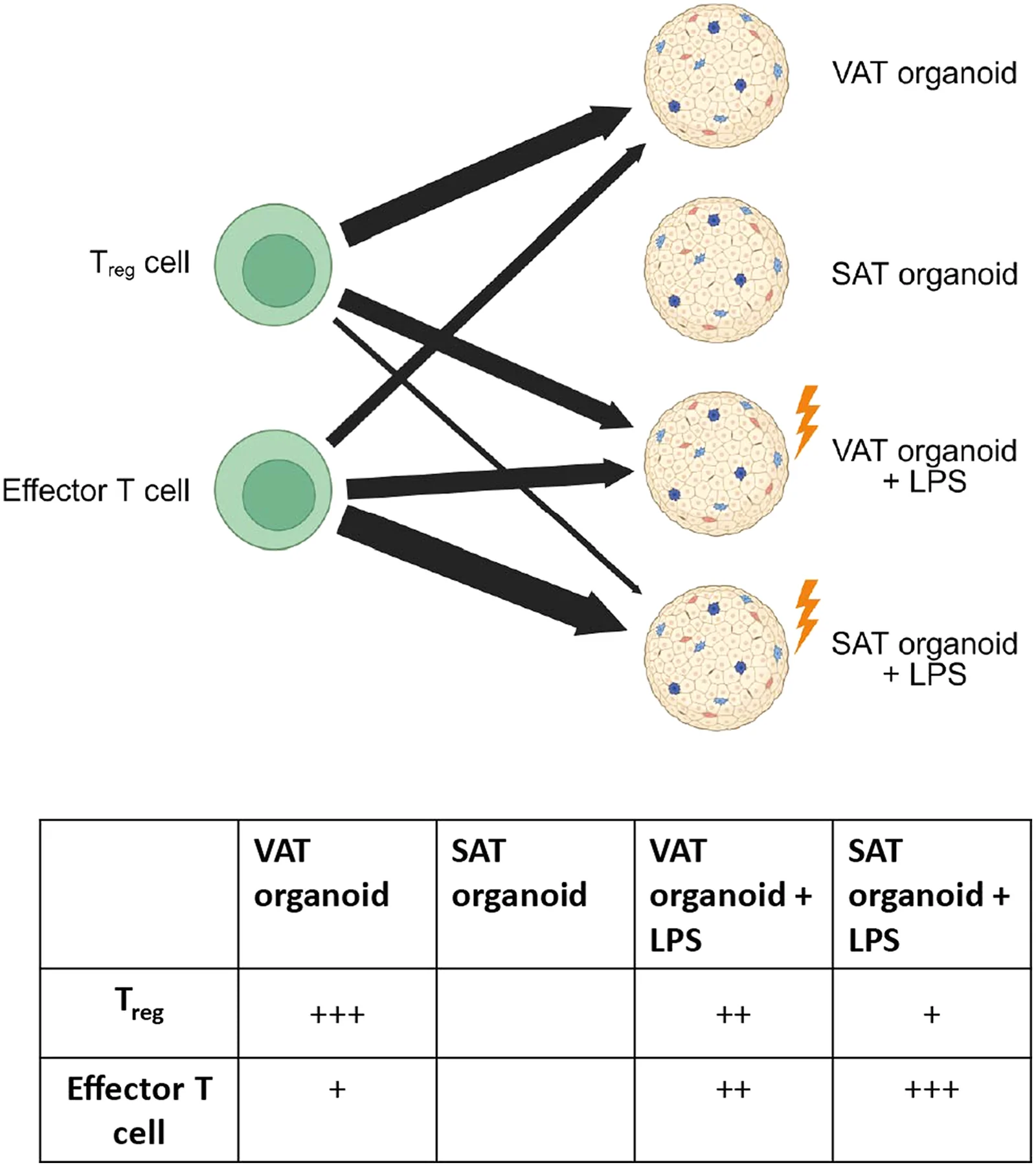
T cells infiltrate adipose tissue organoids after co-cultivation T_reg_ and effector T cells primarily infiltrate unstimulated and LPS-stimulated VAT organoids. LPS stimulation results in an increased T cell infiltration into VAT organoids. Interestingly, T cells only infiltrate SAT organoids upon LPS stimulation. LPS, lipopolysaccharides; SAT, subcutaneous adipose tissue; T_reg_, regulatory T cell; VAT, visceral adipose tissue. Created in BioRender: https://BioRender.com/4bksfd1.

Interestingly, using LPS stimulation to induce inflammatory conditions, we could observe that CD4^+^ T cells infiltrate LPS-stimulated SAT organoids while T cell infiltration is marginally increased in VAT organoids upon LPS stimulation. Therefore, LPS may be used to enhance T cell infiltration rates in both adipose tissue organoid types. We could further demonstrate that LPS-stimulated SAT organoids contain lower frequencies of T_reg_ cells compared with LPS-stimulated VAT organoids. LPS stimulation of organoids may primarily induce the infiltration of effector T cells into adipose tissue organoids (Figure 4).

Currently, we hypothesize that LPS stimulation leads to the activation of Toll-like receptor 4 (TLR4) which is expressed by ASCs, adipocytes, and macrophages within the adipose tissue organoids. Recent studies showed that LPS-stimulation of ASCs and adipocytes results in secretion of pro-inflammatory cytokines such as TNF-α and IL-6.^11^^,^^12^ It was shown that TLR4-activated adipocytes are responsible for most of the secreted TNF-α in inflamed adipose tissue^13^ and saturated fatty acid-derived metabolites from adipocytes may induce pro-inflammatory stimulation of immune cells in a TLR4-independent manner.^14^ It is known that TNF-α-induced C-C motif chemokine ligand 5 (CCL5) expression by adipose stem cells leads to T cell infiltration during obesity.^15^ CCL5 is recognized by T cells via C-C chemokine receptor type 5 (CCR5), so a critical role for the CCL5/CCR5 axis is supposable. How exactly adipocyte activation induces T cell migration and why T_reg_ cells and effector T cells behaved differently will be addressed in further studies.

To date, the model has only been validated in a murine system from male mice. Interestingly, recent findings in mice showed that VAT-T_reg_ populations demonstrate different gene expression depending on the sex.^16^^,^^17^ By comparing adipose tissue organoids from male and female mice, sex difference in adipose tissue immunology can be further studied.

In summary, we established a model that is easily adjustable to fit specific experimental needs. The immune cells co-cultivated with the organoids can be isolated from various genetically altered mice to study the effects of overexpression, downregulation, or deficiency of specific genes. Drug treatment is also readily available to study adipose tissue immunology and to possibly find new therapies for diseases associated with adipose tissue. Furthermore, various methods such as histology, flow cytometry, RNA analysis, and cytokine assays are readily available for this model.^7^ Additionally, the general principle of our adipose tissue organoid may be expanded on human donor cells to generate human adipose tissue organoids containing T cells. Recent studies revealed that SAT organoids can be generated in a similar manner from human donor material.^18^^,^^19^

In conclusion, we herein provide a suitable *ex vivo* tool to analyze adipose tissue-resident T cells from different fat depots under near-physiological conditions. Since adipose tissue-resident T cells, especially VAT T_reg_ cells, are only available in low numbers, our model presents a platform to effectively study the role of T cells in adipose tissue. This system may be used to study the differentiation and migration of T cells into adipose tissue, to analyze the role of VAT-T_reg_ cells in adipose tissue inflammation and insulin resistance, or for screening new therapeutical compounds to ameliorate adipose tissue-related diseases.

### Limitations of the study

Despite the advantages of our adipose tissue organoid model containing T cells, several limitations should be noted. First, the organoids are relatively small, with only 50,000 ASCs seeded per unit, which may restrict scalability and downstream applications. In addition to this, splenocytes are added in excess, which may not accurately reflect physiological cell ratios, and pre-sorting of T cells from splenocytes under sterile conditions might also further improve the assay.

As mentioned within the discussion, our study so far only includes organoids and T cells from male mice, and further studies are necessary with female organoids and T cells.

One limitation is also that we have so far only used LPS to induce inflammation in adipose tissue organoids. The use of TNF-α and IL-1β might be a more physiological inducer of inflammation and will be assessed in further studies.

The development of a corresponding human organoid model would be a valuable next step, enabling the investigation of T cell function in human adipose tissue organoid physiology. Recent studies regarding a small intestinal organoid model showed that tissue-resident T cells may perform better than peripheral blood mononuclear cells (PBMCs) for the establishment of immuno-organoids^20^ and might be a useful cell type.

Limitations also exist for downstream analysis. The low cell number per organoid necessitates pooling multiple organoids to obtain sufficient material for nucleic acid, protein, or cellular component isolation. Flow cytometry requires enzymatic digestion, which can alter surface antigen expression (e.g., CD4 and CD8), thereby potentially biasing the results. Intracellular staining and milder conditions during enzymatic digestion may mitigate this problem.^18^

## Resource availability

### Lead contact

Further information and requests for resources and reagents should be directed to and will be fulfilled by the lead contact, Kim Ohl (kim.ohl@rwth-aachen.de).

### Materials availability

This study did not generate new unique reagents. All unique/stable reagents generated in this study are available from the lead contact with a completed materials transfer agreement.

### Data and code availability

• All relevant data are presented in the manuscript and accompanying supplemental information. No new datasets were generated or deposited during this study.
• All data reported in this paper will be shared from the lead contact upon request.
• This paper does not contain newly generated codes.
• Further information and requests for other items should be directed to the lead contact.

## Acknowledgments

The study was supported by a grant from the Interdisciplinary Center for Clinical Research (to K.O.) within the faculty of Medicine at the 10.13039/501100007210RWTH Aachen University (PTD P07). H.W. was supported by the Jürgen Manchot Foundation and the 10.13039/501100001659Deutsche Forschungsgemeinschaft (German Research Foundation; project WE 2625/4-1). The authors are grateful to Lillia Koop and Judit Turyne Hriczko for technical assistance. Graphical abstract and Figure 4 were created in BioRender: https://BioRender.com/k3os0wt and https://BioRender.com/4bksfd1.

## Author contributions

Conceptualization, K.O., H.W., L.R., S.B.B., T.R., J.B., and K.T.; methodology, C.K., Y.M., S.H.S., C.T.N., and C.L.; writing – original draft, C.K., H.W., Y.M., and K.O.; writing – review and editing, C.K., S.H.S., C.T.N., T.R., J.B., and S.B.B.; project administration, K.O. and H.W.; supervision, K.O., H.W., L.R., and K.T. All authors have read and approved the final version of the manuscript.

## Declaration of interests

The authors declare no competing interests.

## STAR★Methods

### Key resources table

**Table:** 

REAGENT or RESOURCE	SOURCE	IDENTIFIER
**Antibodies**		

monoclonal anti-CD3 (APC conjugated, clone 145-2C11)	Thermo Fisher Scientific	Cat# 17–0031–82; RRID:AB_10756850
monoclonal anti-CD4 (eFluor™ 450 conjugated, clone RM4-5)	Thermo Fisher Scientific	Cat# 48–0042–82; RRID:AB_1272231
monoclonal anti-CD8 (PE-Cyanine7 conjugated, clone 53–6.7)	Thermo Fisher Scientific	Cat# 25–0081–82; RRID:AB_1272198
monoclonal anti-CD45 (APC-eFluor 780 conjugated, clone 30-F11)	Thermo Fisher Scientific	Cat# 47–0451–82; RRID:AB_1548781
anti-CD69-PE (clone H1.2F3)	Thermo Fisher Scientific	Cat# 12–0691–82; RRID:AB_10007657
Fixable Viability Dye eFluor 520	Thermo Fisher Scientific	Cat# 65–0867–14

**Biological samples**		

Murine visceral adipose tissue (VAT, gonadal fat)	This manuscript	Male C57BL/6 mice, 7–9 weeks RRID:MGI:2159769
Murine subcutaneous adipose tissue (SAT, inguinal)	This manuscript	Male C57BL/6 mice, 7–9 weeks, RRID:MGI:2159769
Murine splenocytes	This manuscript	Male FOXP3-Cre-ROSA-RFP mice, own breeding

**Chemicals, peptides, and recombinant proteins**		

Collagenase II	Pan Biotech (Worthington – USA origin)	Cat# LS0004177
Liberase TL	Roche	Cat# 05401020001
Lipopolysaccharide (LPS from E. coli)	Sigma-Aldrich	Cat# L2880
IBMX (3-isobutyl-1-methylxanthine)	Cayman Chemicals	Cat# 13347
Rosiglitazone	Cayman Chemicals	Cat# 71740
Dexamethasone	Sigma-Aldrich	Cat# D4902
Insulin (recombinant human)	Sigma-Aldrich	Cat# I9278
T3 (3,3′,5-Triiodo-L-thyronine sodium salt)	Sigma-Aldrich	Cat# T6397
L-Ascorbic acid	Sigma-Aldrich	Cat# A4034
D-Biotin	Sigma-Aldrich	Cat# B4639
Pantothenic acid (calcium salt)	Hycultec	Cat# HY-N0681
Amphotericin B	Gibco	Cat# 15290–026
Trypsin EDTA	Gibco	Cat# 25300–054
DMSO (Dimethyl sulfoxide)	Carl Roth	Cat# A994.2
Sodium Hydroxide (NaOH)	Sigma-Aldrich	Cat# 1.06498
Ethanol	Carl Roth	Cat# 9065.4
DMEM (4.5 g/L glucose, with Na-pyruvate, L-Glutamine)	PAN Biotech	Cat# P04-04510
Fetal Calf Serum (FCS, heat inactivated, qualified)	Gibco	Cat# A5256801
Penicillin/Streptomycin (P/S)	Gibco	Cat# 15140–122
Bovine Serum Albumin (BSA, fatty acid free)	Sigma-Aldrich	Cat# A8806
RBC lysis buffer	Invitrogen	Cat#00–4300–54

**Critical commercial assays**		

IL-6 ELISA kit (mouse)	Biolegend	Cat# 431304
TNF-α ELISA kit (mouse)	Biolegend	Cat# 430904
eBioscience™ Foxp3/Transcription Factor Staining Buffer Set	Invitrogen	Cat# 00–5523–00

**Experimental models: Organisms/strains**		

Mouse: C57BL/6 (male, 7–9 weeks old)	Internal breeding	RRID:MGI:2159769
Mouse: Rosa26-RFP x Foxp3-Cre (FOXP3 reporter line, male)	Tobias Bopp (Institute of Immunology, JGU Mainz, Germany) and internal breeding.^21^	–

**Software and algorithms**		

FlowJo v10.8	BD Biosciences	https://www.flowjo.com; RRID:SCR_008520
GraphPad Prism v10	GraphPad Software	https://www.graphpad.com; RRID:SCR_002798
BioRender	BioRender	https://biorender.com; RRID:SCR_018361

**Other**		

96-well U-bottom ultra-low attachment plate (Nunclon Sphera)	Thermo Scientific	Cat# 174925
Freezing container, Nalgene® Mr. Frosty	Sigma-Aldrich	Cat# C1562

### Experimental model and study participant details

#### Mice

All animal experiments were performed in compliance with the German animal protection law (TierSchG) and approved by the local animal welfare committees and the Landesamt für Verbraucherschutz und Ernährung (LAVE), North-Rhine Westphalia (internal numbers: 30277A4, 50199A4). All procedures were performed in accordance with the relevant guidelines and regulations laid down by the national authorities of the LANUV and fulfilled the criteria of the German administrative panel on laboratory animal care. Mice were housed under standard laboratory conditions with a 12-h light/dark cycle, controlled temperature (22 ± 2°C), and *ad libitum* access to standard chow and water under specific pathogen-free conditions.

This study was performed entirely with male mice. Results should be confirmed in female mice. Male C57BL/6 mice aged 7–9 weeks were used for adipose tissue isolation and adipose stromal/progenitor cell derivation. Male FOXP3^CRE^ROSA^RFP^ reporter mice (Rosa26-RFP x Foxp3-Cre) were used for splenocyte isolation. The reporter line was kindly provided by Tobias Bopp (Institute of Immunology, Johannes Gutenberg University, Mainz, Germany).

### Method details

#### Isolation of murine adipose tissue

Mice were sacrificed by cervical dislocation. Visceral adipose tissue (VAT) was harvested from the gonadal fat depot surrounding the testis. Subcutaneous adipose tissue (SAT) was isolated from the posterior inguinal region around the hind limbs. For each experiment, adipose tissue from two individual mice was pooled.

#### Adipose tissue organoid generation

Adipose tissue samples were collected in ice-cold PBS and minced with scissors. Digestion was performed with collagenase II (2 mg/ml) and BSA (3 mg/ml) in DMEM at 37°C for 20 min. After filtering through a 100 μm mesh strainer, samples were centrifuged and stromal vascular fraction (SVF) pellet was resuspended in growth medium (DMEM +10% FCS +1% P/S) at 37°C, 5% CO_2_ in a humidified incubator to initiate 2D pre-culture. Medium was changed every 2–3 days and cells were passaged at approximately 80% confluence. Subsequently, adipose tissue organoids were generated from 2D-expanded ASCs using ultra-low attachment 96-well U-bottom plates to facilitate spheroid self-assembly. To this end, cell detachment was performed with pre-warmed Trypsin/EDTA and 50,000 cells were added to each well of a 96-well U-bottom ultra-low attachment plate (Nunclon Sphera). After 2–3 days, growth medium was replaced by induction medium (25 mL growth medium +2.5 μL insulin (10 mg/mL) + 10 μL dexamethasone (1 mg/mL in EtOH) + 0.4 μL T3/DMEM +2.5 μL rosiglitazone (10 mM in DMSO) + 25 μL ABP (L-ascorbic acid (1 g in 10 mL ddH_2_O), d-biotin (4.89 mg in 0.5 mL 1 M NaOH), and pantothenic acid (81 mg in 9.5 mL ddH_2_O.)). After 2 days induction medium was replaced by differentiation medium (50 mL growth medium + 5 μL insulin (10 mg/mL) + 0.8 μL T3/DMEM (1 mg T3 dissolved in 0.5 mL 1 M NaOH), then diluted to 25 mL with DMEM + 50 μL ABP). Medium was replaced every 2–3 days. After 6 days of differentiation, organoids are considered fully differentiated and ready for co-culture with splenocytes or other downstream applications.

#### LPS stimulation of adipose tissue organoids

To model pro-inflammatory conditions and enhance T cell infiltration, organoids were optionally stimulated with LPS prior to co-culture. To this end, medium was aspirated and fully differentiated organoids (day 11+). were incubated with 10 ng/mL LPS containing medium for 1 h. After 1 h of incubation LPS-containing medium was replaced.

#### Isolation of murine splenocytes

Splenocytes were isolated fresh and used immediately for co-culture. Harvested spleen from euthanized mouse were pressed through a 0.45 µm strainer using the plunger end of a syringe to obtain single cell suspensions. After centrifugation, cells were incubated with RBC lysis buffer for 3 min to remove red blood cells and subsequently washed with PBS containing 0.5% BSA. Cells were counted and resuspended in growth medium at a final concentration of 5 × 10^6^ cells/mL for co-culture.

#### Organoid-splenocyte co-culture

Fully differentiated adipose tissue organoids were co-cultivated with freshly isolated splenocytes to introduce T cells. DMEM supplemented with 10% (v/v) heat-inactivated FCS and 1% (v/v) penicillin/streptomycin was used as standard growth medium. Therefore, 1 × 10^6^ splenocytes were added per well and cocultured for 2 days. As controls monocultures of splenocytes and organoids were incubated separately under identical conditions.

#### Flow cytometry

Organoids were separated from non-infiltrating splenocytes, washed, and enzymatically digested to generate single-cell suspensions. After two washing steps with PBS, organoids were digested with Liberase TL (10 μL, 4 U/mL working stock) for 20 min at 37°C. Digestion was stopped by adding PBS/BSA 0.5%. Cell surface staining was done in 0.5% BSA in PBS or 30 min on ice. The intracellular staining was performed by fixation and permeabilization using the eBioscience Foxp3/Transcription Factor Staining Buffer Set (Invitrogen) according to manufactures instructions. Prior to Flow cytometric analysis cells were added to tubes with 35 μm cell strainer caps to remove debris and aggregates.

Samples were analyzed on a flow cytometer equipped with appropriate lasers and filters for eFluor 520, PE, PE-Cyanine7, eFluor 450, APC, and APC-eFluor 780 detection. Compensation controls were prepared using splenocyte monocultures or supernatant cells from co-cultures. A representative gating strategy is shown in Figures 1B and S1A): **Lymphocyte gate:** FSC-A vs. SSC-A to identify lymphocyte population, **Singlet gate:** FSC-H vs. FSC-A to exclude doublets, **Viable cells:** SSC-A vs. Viability Dye-eFluor 520 to gate on live (dye-negative) cells, **T cells:** CD45-APC-eFluor 780+ (immune cells) and CD3-APC+ (T cells) to identify infiltrating T lymphocytes, **T**_**reg**_ **cells** (when using FOXP3-RFP reporter mice): RFP expression (PE channel) within CD3^+^ gate to identify FOXP3+ regulatory T cells, **CD4**^**+**^ **T cells**: CD4-eFluor 450+ and CD8-PE Cyanine7-expression within CD3^+^ gate to identify CD4^+^ T cells, **CD8**^**+**^ **T cells**: CD4-eFluor 450- and CD8-PE Cyanine7+ expression within CD3^+^ gate to identify CD8^+^ T cells, **CD69**^+^ T cells: CD69-PE+ expression within CD3-APC gate. Data were analyzed using FlowJo software (or FCS Express, or equivalent). At least 4 organoid replicates were analyzed per condition. For splenocyte control samples, 1 × 10^6^ cells were analyzed per sample.

#### Cytokine quantification by ELISA

Supernatants collected from co-cultures (step 2 of organoid preparation protocol) were stored at −80°C until analyzed and IL-6 and TNF-α levels were determined using mouse-specific ELISA kits according to the manufacturer’s instructions.

#### Histology

H&E stainings were performed as described in previous work.^21^ Briefly, 4 μm paraffin sections from the fixed organoids were cut serially, mounted onto glass slides, and deparaffinized. Blinded histological scoring was performed using a standard microscope.

### Quantification and statistical analysis

**Sample sizes and replicates: Organoid co-cultures:** 4 organoids were pooled per sample for flow cytometric analysis. Each experimental condition was performed with at least 3 biological replicates (*n* ≥ 3). **Splenocyte controls:** 1 × 10^6^ cells were analyzed per sample for compensation and gating reference. **ELISA assays:** *N* = 4 biological replicates per condition. **Independent experiments:** Key findings were validated across at least 3 independent experiments using different batches of organoids and freshly isolated splenocytes.

#### Statistical analysis

All statistical analyses were performed using GraphPad Prism software (version 10.6.1) on untransformed percentage values. Data are presented as mean ± standard error of the mean (SEM) or as individual data points with mean, as indicated in figure legends. Data that was identified as significant outliers were excluded. All experiments were independently replicated at least three times with consistent results. All statistical details of an experiment can be found in the figure legends.

**Statistical tests: Kruskal-Wallis test:** For statistical comparison of the T_reg_ frequencies a non-parametric Kruskal-Wallis test was used followed by Dunn’s multiple-comparison test. (Figures 1E, 2D, and 3D). **two-way ANOVA:** two-way ANOVA was used to compare samples from organoid co-cultures from adipose tissue types (VAT vs. SAT) and co-culture settings (control vs. +splenocytes) followed by a Tukey’s post hoc test. (Figure 2C). **Three-way ANOVA:** Three-way ANOVA was used to compare samples between the organoid type (VAT vs. SAT), co-culture condition (control vs. +splenocytes), and LPS stimulation (unstimulated vs. +LPS). Post hoc pairwise comparisons were performed with Sidak’s correction for multiple testing (Figure 3B). **Unpaired comparisons:** A two-tailed unpaired Student’s *t* test was used to compare independent groups (e.g., unstimulated vs. LPS-stimulated organoids; Figures S1C and S1D). **Significance thresholds:** Statistical significance was defined as *p* < 0.05. Exact *p* values or significance levels are indicated in figures as: ∗ <0.05, ∗∗ <0.01, ∗∗∗ <0.001, ∗∗∗∗ <0.0001, or ns (not significant, *p* ≥ 0.05).
