# Supplementary material for: Generation of T cell containing adipose tissue organoids
Source: iScience. 2026 Sep 3;29(9):117429. doi: 10.1016/j.isci.2026.117429 (PMC13571656; doi:10.1016/j.isci.2026.117429)
Supplement: Supplementary file 1 [file mmc1.pdf]

iScience, Volume *29*

Supplemental information

Generation of T cell

containing adipose tissue organoids

Christian Kühne, Yasmin Majlesain, Sudheendra Hebbar Subramanyam, Christopher T. Neullens, Charlotte Lahrtz, Tim Ruhl, Justus Beier, Sabrina B. Bennstein, Lothar Rink, Klaus Tenbrock, Heike Weighardt, and Kim Ohl

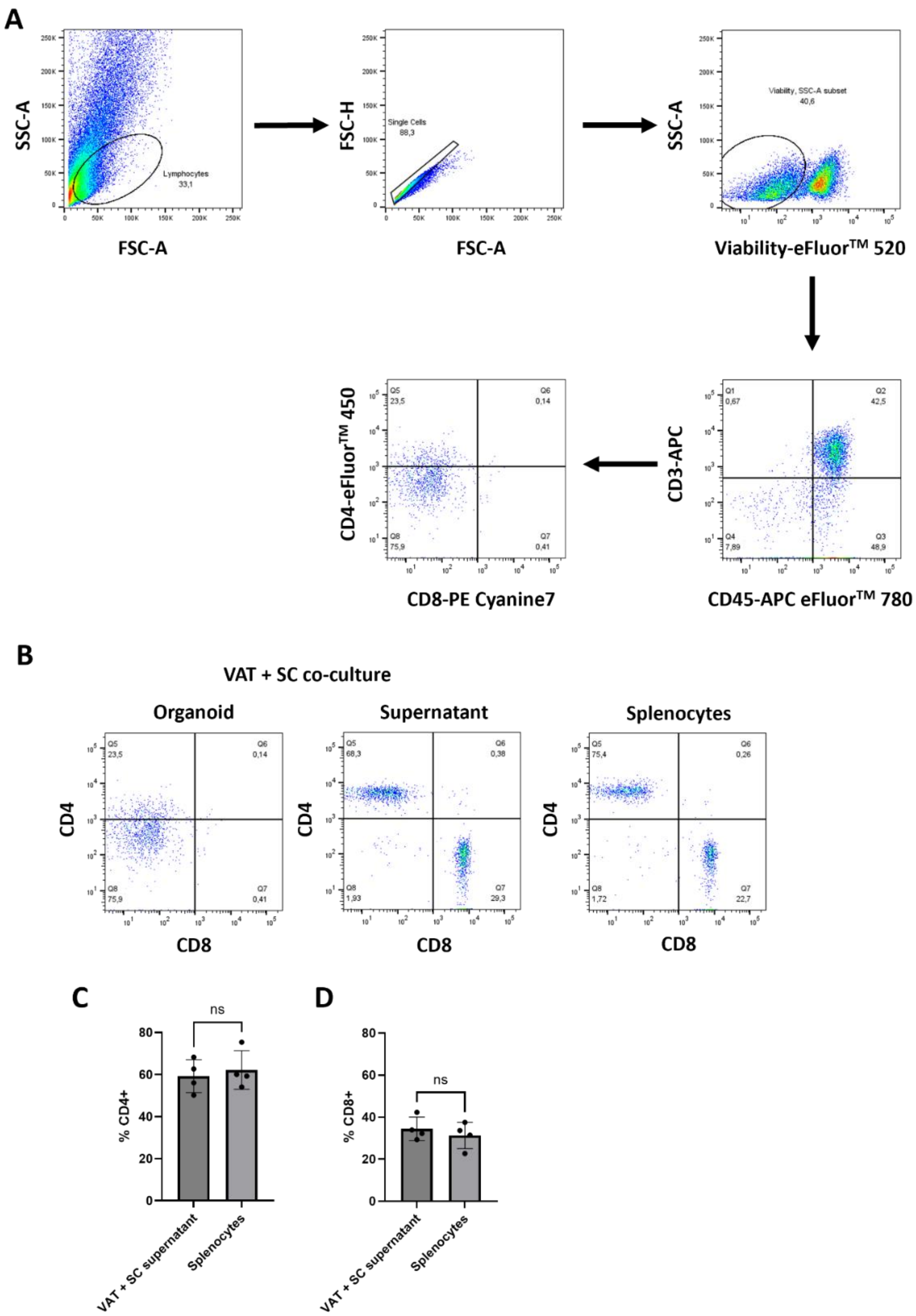

Figure S1: Adipose tissue organoid co-cultures contain mainly CD4+ (A) Gating strategy for the identification of CD4+ and CD8+ T lymphocytes in an adipose tissue organoid model. After the differentiation into adipose tissue organoids and the co-cultivation with murine SC was completed, organoids were separated from their supernatants. Both fractions were isolated, washed and digested for subsequent flow cytometric analysis. Splenocyte monoculture was used as control. Staining was performed with Viability Dye eFluor^{TM} 520 (FITC) (1:1000), anti-mouse CD45 APC eFluor^{TM} 780 (APC-Cy7) (1:100), anti-mouse CD3 APC antibodies (1:100), anti-mouse CD4 eFluor^{TM} 450 (Pacific Blue) (1:100), and anti-mouse CD8 PE-Cyanine7 (1:100). Depicted is a co-culture of VAT organoids with splenocytes from FOXP3^{CRE}ROSA^{RFP} mice (VAT+ SC). For each sample 4 organoids were used. 4 replicates were measured per sample. (B) Representative populations of CD4+ and

CD8+ T cells in SC monoculture and in the organoid and supernatant fraction of VAT + SC co-cultures and in splenocyte monocultures. (C) CD4+ T cell frequency was measured as percentage of CD45+ CD3+ T cells. CD4+ T cell ratios were compared between VAT + SC co-culture supernatants and splenocytes only using an unpaired t-test. (D) CD8+ T cell frequency was measured as percentage of CD45+ CD3+ T cells. CD8+ T cell ratios were compared between VAT + SC co-culture supernatants and splenocytes only using an unpaired t-test. GraphPad Prism (v10.6.1.) was used for all analyses. Data is presented as mean ± SEM and the indicated data points. Statistical significance is represented as ns (not significant). SC: splenocytes. VAT: visceral adipose tissue.

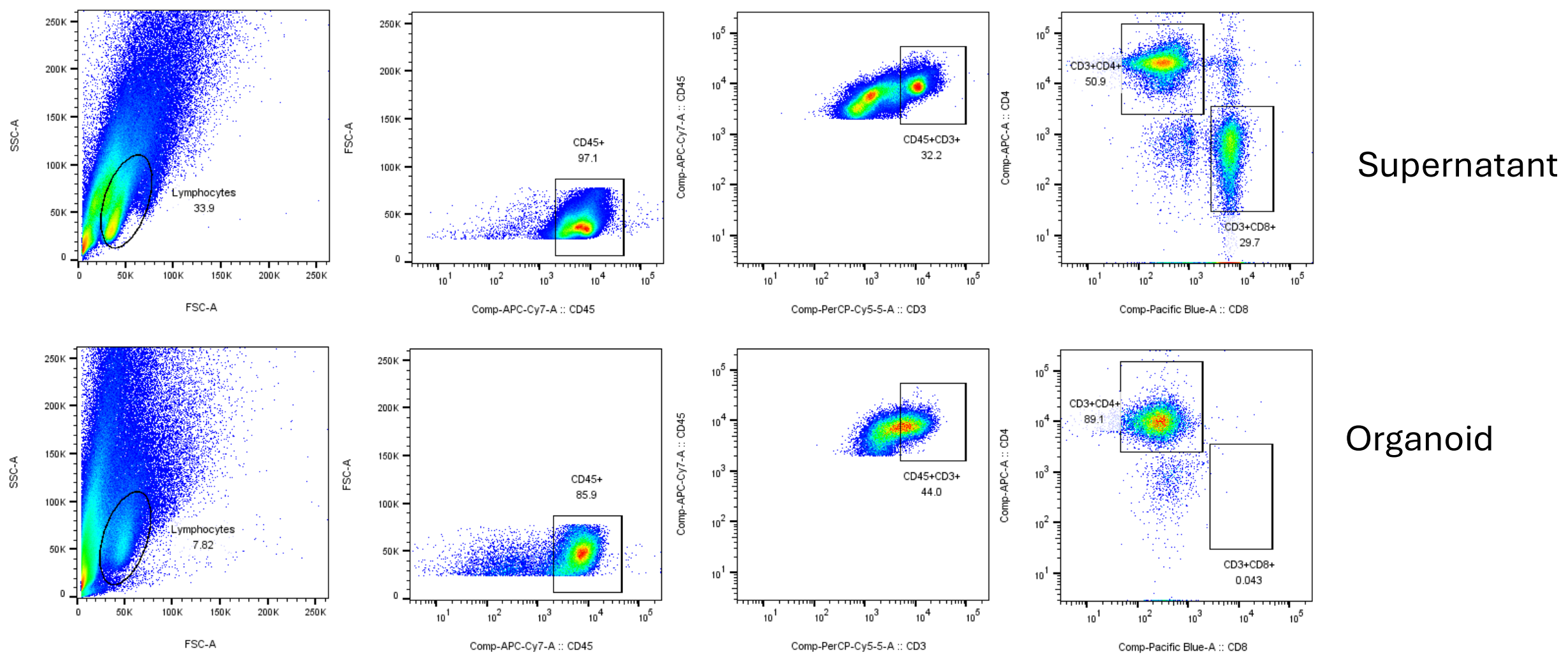

Figure S2: LPS stimulated adipose tissue organoid co-cultures contain mainly CD4+ T cells. A) Gating strategy for the identification of CD4+ and CD8+ T lymphocytes in an adipose tissue organoid model after LPS treatment. After the differentiation into adipose tissue organoids and the co-cultivation with murine SC was completed, organoids were separated from their supernatants. Both fractions were isolated, washed and digested for subsequent flow cytometric analysis. Splenocyte monoculture was used as control. Cells were fixed and permeabilized using Foxp3 Intracellular Staining Kit according to the manufacturer's instructions, with a fixation step of 30 minutes at 4°C. Staining was performed with anti-mouse CD45 APC eFluor™ 780 (APC-Cy7) (1:100), anti-mouse CD3 APC antibodies (1:100), anti-mouse CD4 eFluor™ 450 (Pacific Blue) (1:100), and anti-mouse CD8 PE-Cyanine7 (1:100). Depicted is a co-culture of VAT organoids with splenocytes (VAT+ SC).
